# Enhancement of recombinant human IL-24 (rhIL-24) protein production from site-specific integrated engineered CHO cells by sodium butyrate treatment

**DOI:** 10.1007/s00449-022-02801-0

**Published:** 2022-10-25

**Authors:** Jilei Hua, Hanli Xu, Yao Zhang, Jianlin Ge, Mengzhe Liu, Yuqi Wang, Yuexian Wei, Yinan Shi, LingLing Hou, Hong Jiang

**Affiliations:** 1 College of Life Science and Bioengineering, Beijing Jiaotong University, No. 3 Shangyuancun, Beijing, 100044 People’s Republic of China; 2 National Key Laboratory of Biochemical Engineering, Institute of Process Engineering, Chinese Academy of Sciences, Beijing, 100190 People’s Republic of China; 3 Institute of Acupuncture and Moxibustion, China Academy of Chinese Medical Sciences, Beijing, 100700 People’s Republic of China

**Keywords:** rhIL-24, Serum-free culture, Suspension culture, CHO cells, Sodium butyrate

## Abstract

Interleukin-24 (IL-24) has specific inhibitory effects on the proliferation of various tumor cells with almost no toxicity to normal cells. The antitumor activity of recombinant human IL-24 protein produced in mammalian cells is much higher than that of bacteria, but its expression level is extremely low. Sodium butyrate (NaBu) was utilized as a media additive to increase protein expression in Chinese hamster ovary cells. The site-specific integrated engineered cells FCHO/IL-24 were treated with NaBu under different culture conditions (10% and 0.5% serum adherent culture, 0.5% serum suspension culture). First, 3 days of 1 mmol/L NaBu treatment significantly increased rhIL-24 expression level in FCHO/IL-24 cells by 119.94 ± 1.5% (**p < 0.01), 57.49 ± 2.4% (**p < 0.01), and 20.17 ± 3.03% (*p < 0.05) under the above culture conditions. Second, NaBu has a time- and dose-dependent inhibitory effect on FCHO/IL-24 proliferation and induces G0/G1 phase arrest. Under 10% and 0.5% serum adherent culture, G0/G1 phase cells were increased by 11.3 ± 0.5% (**p < 0.01) and 15.0 ± 2.6% (**p < 0.01), respectively. No induction of apoptosis was observed under a high dosage of NaBu treatment. These results suggest that NaBu increases rhIL-24 secretion via inhibiting cell cycle progression, thereby trapping cells in the highly productive G0/G1 phase. Finally, with increasing NaBu dose, glucose concentration increased (**p < 0.01) while lactic acid and ammonia concentrations reduced significantly (**p < 0.01) in 10% and 0.5% serum adherent culture supernatant. RNA-seq showed that NaBu treatment affected multiple tumor and immune-related pathways. In conclusion, NaBu treatment dramatically promoted rhIL-24 production in engineered FCHO/IL-24 cells by altering downstream pathways and inducing G0/G1 cell arrest with little effect on apoptosis.

## Introduction

In the 1980s, recombinant DNA technology led to the approvals of the first recombinant protein drug Humulin in 1982, and muromonab—the first monoclonal antibody used as a therapeutic agent in 1985. These approvals launched the third wave of the biopharmaceutical industry boom that is still fast-growing at present, of which recombinant therapeutic proteins remain pillars in translational medicine development and application [1, 2]. Among different systems of recombinant protein production, mammalian expression systems are generally preferred, especially for large, complex molecules that require specific posttranslational modifications (most notably glycosylation) that is executed correctly only in mammalian expression systems. Additionally, in engineered mammalian cell lines, most recombinant proteins can be secreted and directly purified from cell culture supernatant without cell lysis, as required in bacterial systems [3]. Over the past few decades, Chinese hamster ovary (CHO) cells represent the most commonly used production cell line for therapeutic proteins [2]. We previously engineered a stable site-specific-integrated cell line, Flp-In^TM^CHO/IL-24 (FCHO/IL-24), to express recombinant human IL-24 (rhIL-24) using the Flp-In system. Under the same cell density and culture time, the expression level of rhIL-24 in FCHO/IL-24 cell line was higher than that of three randomly integrated human embryonic kidney 293 (HEK293) cell lines (HEK293/pSecTag2A-IL-24, HEK293/pcDNA3.1myc/His-IL-24 and HEK293/pCEP4-IL-24) [4].

Improving recombinant protein yield is one of the most important considerations in converting promising therapeutic protein candidates into products ready for clinical trial and the market. Among the few known strategies, such as host cell engineering, vector improvement, and serum-free suspension culture optimization, additives with mild toxicity have been considered as the most convenient way to increase protein production [5] and sodium butyrate (NaBu) is the additive best known for this purpose. Butyrate is a short fatty acid chain and NaBu acts as a noncompetitive inhibitor of a histone deacetylase to regulate the chromatin structure of mammalian cells, halt DNA synthesis, arrest cell proliferation, alter cell morphology and regulate gene expression [6, 7]. It could significantly increase the expression level of many recombinant proteins, especially glycosylated proteins in CHO cells, such as interferon-γ (IFN-γ) [8], tissular plasminogen activator (t-PA) [9], erthyropoietin (EPO) [10], von Willebrand factor (vWF) [10], thrombopoietin (TPO) [11], prolactin [12], thyrotropin [13], recombinant IgG [14], insulin, GLUT1, the group-I metabotropic glutamate receptor and PKC [15], etc. NaBu can stimulate CHO cells [9] and human endothelial cells [16] to increase the expression of t-PA under different culture conditions (adherent culture with 20% human serum [16] and serum-free suspension culture [9]). NaBu treatment can improve the specific productivity of recombinant human EPO in human kidney fibrosarcoma cell line HT1080 [17]. In addition, NaBu can increase the molar ratio of total sialic acids to recombinant human IFN-γ produced by engineered CHO cells [8] and modify oligosaccharide content of glycoproteins in various expression systems [17]. However, NaBu treatment can also lead to a net reduction of IFN-β levels due to higher specific productivity (IFN-β units/cell/day) and reduced growth rates [18].

IL-24, a protein consisting of 206 amino acid residues, was discovered in 1995 by Paul Fisher [19] and regarded as the “magic bullet” for treating cancers due to its broad-spectrum and specific antitumor activity [20]. As a glycoprotein with three glycosylation sites at Asn (85, 99, 126) [21], the secreted rhIL-24 purified from mammalian cell culture supernatant has the highest antitumor activity compared to rhIL-24 from other host cell systems, including *E. coli*, yeast and insects [4, 22]. The yields of rhIL-24 in eukaryotic cell systems are much lower than in prokaryotic systems, so systematic optimization is essential to allow mass production. We recently published the successful adaptation of FCHO/IL-24 cells from an adherent culture in DMEM/F12 with 10% serum, to a suspension culture in serum-free medium EdenTM-B300S [23]. However, while cutting serum from culture media provided benefits such as lowering the risk of contamination and production cost, it dramatically reduced available nutrients which negatively impacted rhIL-24 production. It was reported that NaBu was able to induce the endongous IL-24 mRNA expression level in human malignant melanoma cell line A375 [24], therefore, we treated adapted FCHO/IL-24 cells with various concentrations of NaBu under different culture conditions to further optimize rhIL-24 production. The NaBu treated-FCHO/IL-24 cells showed significantly improved rhIL-24 secretion, along with halted cell proliferation and G0/G1 phase arrest. Thus, NaBu can be used as an additive for optimizing serum-free suspension medium to improve rhIL-24 yield in an engineered cell line for mass production and therapeutic applications.

## Results

### NaBu inhibited proliferation of engineered cells FCHO/IL-24 in vitro

**Fig. 1:**
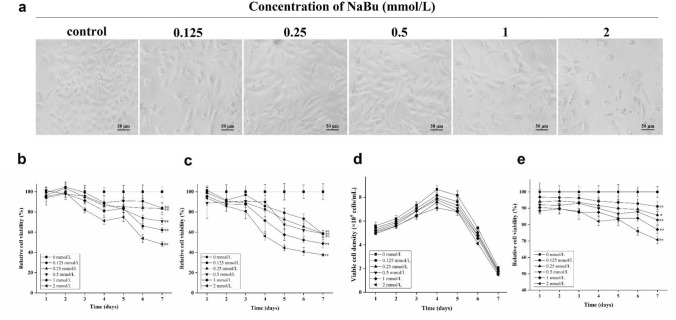
NaBu inhibits FCHO/IL-24 cell proliferation under different culture conditions. **a** Under 10% serum adherent culture condition, cells showed increasingly abnormal morphology and lower cell density with higher NaBu concentration (0, 0.125, 0.25, 0.5, 1, and 2 mmol/L) on day 3, scale bar = 50 μm. **b**, **c** The relative cell viabilities after being treated with different concentrations of NaBu for 7 days under adherent culture with 10% serum and 0.5% serum, respectively. 650 and 2000 adapted cells were seeded in each well of the 96-well plate in 200 μL DMEM/F12 with 10% and 0.5% serum, respectively. The cell viability was analyzed by MTT **e**veryday, and the control groups were not treated by NaBu. **d**, **e** Under 0.5% serum suspension culture the cell numbers and relative cell viabilities decreased evidently with NaBu treatment. The adapted cells were suspension cultured (seeding density 5 × 10^5^ cells/mL, 20 mL total) in DMEM/F12 with 0.5% serum at 119 rpm. Viable cells were counted each day after taking cell mixture and staining cells with tryphan blue. Fresh media was added to make up lost volume due to taking samples for tryphan blue staining

NaBu can not only promote the expression and secretion of recombinant protein but also inhibit cell proliferation in CHO cells [8, 11, 12, 14]. The adapted FCHO/IL-24 cells were adherent cultured in DMEM/F12 with 10% serum [23] and treated with different concentrations of NaBu (0, 0.125, 0.25, 0.5, 1 and 2 mmol/L) for 7 days. As shown in Fig. 1a, with increasing NaBu concentration, cell density and morphology changed drastically after 3 days of treatment. Compared to lower dosage and untreated control groups, there were more dead cells at high concentrations of NaBu (1 and 2 mmol/L). Relative cell viability decreased with increasing NaBu dosage and treatment time (Fig. 1b). Similar results were observed when the cells were adherent cultured in DMEM/F12 with 0.5% serum (Fig. 1c). When cultured under 0.5% serum suspension culture conditions, the cells entered exponential growth phase on the 2nd day, peaked on the 4th day, and then died rapidly regardless of NaBu dose (Fig. 1d). Similar to cells under adherent culture conditions, NaBu also inhibited relative cell viability of FCHO/IL-24 cells in suspension culture (Fig. 1e). For example, after 3 days of 1 mmol/L NaBu treatment, inhibition of cell proliferation were 9 ± 3% (*p < 0.05), 13 ± 2% (**p < 0.01) and 12 ± 2% (**p < 0.01), respectively, under 10% serum adherent culture, 0.5% serum adherent culture and 0.5% serum suspension culture. The results showed that NaBu had a dose- and time-dependent inhibitory effect on FCHO/IL-24 cell proliferation in vitro under adherent and suspension culture with high/low serum content.

### NaBu promoted the secretion of rhIL-24 by FCHO/IL-24 cells

**Fig. 2:**
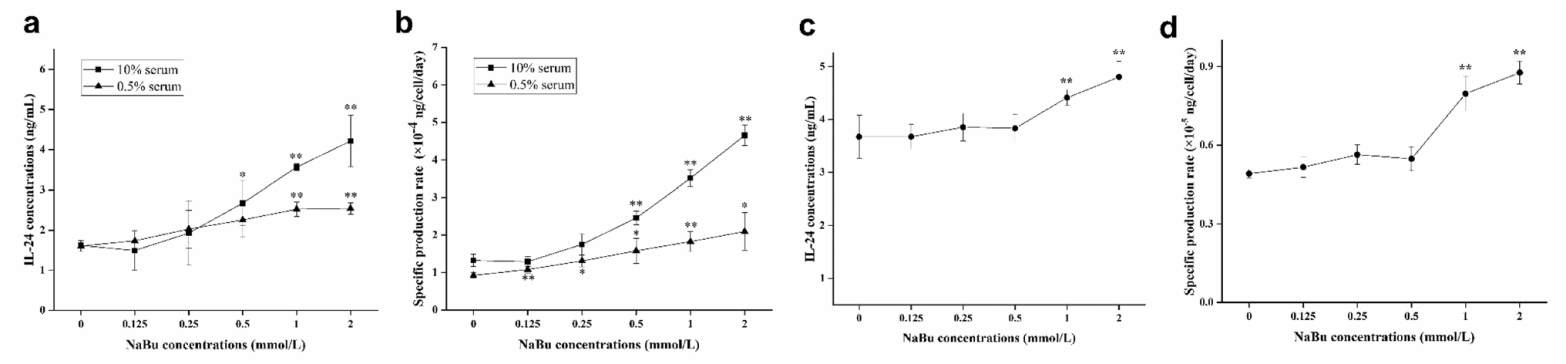
NaBu promoted rhIL-24 expression level in FCHO/IL-24 cells under different culture conditions. **a**, **b** Under 10% and 0.5% serum adherent culture, compared to untreated control group, different concentrations of NaBu (0.125, 0.25, 0.5, 1, and 2 mmol/L) significantly increased both overall rhIL-24 concentrations and qP (**p < 0.01). 650 and 1500 cells were seeded in each well of 96-well plate in 200 μL DMEM/F12 with 10% and 0.5% serum, respectively. **c**, **d** Under 0.5% serum suspension culture, 3 days of high concentration NaBu treatment significantly increased rhIL-24 expression level in cell culture supernatant and qP compared to untreated control group (**p < 0.01). The adapted cells were suspension cultured (seed density 5 × 10^5^ cells/mL, 20 mL total) in DMEM/F12 with 0.5% serum at 119 rpm

To determine the effect of NaBu on rhIL-24 secretion in adapted FCHO/IL-24 cells, rhIL-24 concentration was tested under different culture conditions (10% and 0.5% serum adherent culture, 0.5% serum suspension culture) by ELISA after 3 days of NaBu treatment. Although cell proliferation was inhibited by NaBu, rhIL-24 expression significantly increased. With different NaBu treatment dosages (0, 0.125, 0.25, 0.5, 1, and 2 mmol/L), rhIL-24 concentrations in 10% serum were 1.62 ± 0.05, 1.50 ± 0.50, 1.93 ± 0.80, 2.67 ± 0.55, 3.56 ± 0.09 and 4.21 ± 0.65 ng/mL, respectively (Fig. 2a). Similar trend was observed in adherent cultured cells with 0.5% serum, though high NaBu dose did not significantly increase IL-24 production overall, with an average value of 2.34 ± 0.17 ng/mL (Fig. 2a). Notably, while total cell number decreased, productivity per cell (qP) increased with higher NaBu concentrations, consistent with a previous observation that NaBu inhibits cell proliferation at higher dosages. On the 3rd day, in both 10% serum and 0.5% serum groups, rhIL-24 concentration and qP were the highest with 2 mmol/L NaBu treatment (Fig. 2a, b), at the expense of 18 ± 4% and 20 ± 5% inhibition of cell proliferation (Fig. 1b, c), respectively. When NaBu concentration was 1 mmol/L, cell proliferation was slowed by 9 ± 3% and 13 ± 2%, respectively, but rhIL-24 expression and qP did not decrease. Compared to cells cultured with 10% serum, the qP values of cells in 0.5% serum were much lower at the same NaBu treatment concentrations, which were consistent with our previous results [23]. Based on the above results, we concluded that for adherent culture in 10% or 0.5% serum, the most suitable NaBu treatment was 1 mmol/L for 3 days to allow for both high relative cell viability and rhIL-24 qP.

Under 0.5% serum suspension culture and 3 days of NaBu treatment (0, 0.125, 0.25, 0.5, 1, and 2 mmol/L), rhIL-24 concentrations in cell culture supernatant were 3.67 ± 0.41, 3.67 ± 0.23, 3.86 ± 0.26, 3.83 ± 0.26, 4.39 ± 0.15, 4.80 ± 0.30 ng/mL, respectively (Fig. 2c). The trend of qP highly similar to that of overall rhIL-24 concentration, both of which increased evidently when NaBu treatment was at least 1 mmol/L or higher. Given that the proliferation rate was comparable between 1 mmol/L and 2 mmol/L NaBu treatment groups (both 12 ± 1%), treatment regimen of 2 mmol/L NaBu for 3 days was selected for future experiments under 0.5% serum suspension culture condition.

### NaBu-induced G0/G1 phase arrest in FCHO/IL-24 cells

**Fig. 3:**
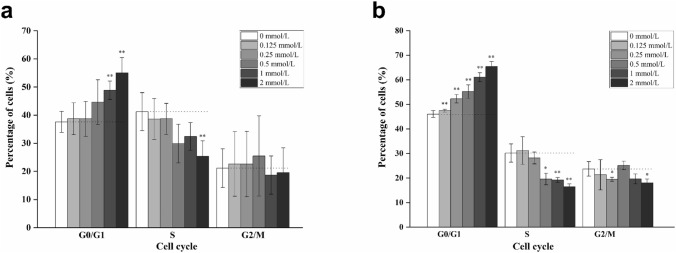
NaBu induced FCHO/IL-24 cells G0/G1 phase arrest. **a** The adapted FCHO/IL-24 cells (1 × 10^5^ cells/well in 6-well plate) were adherent cultured in 10% serum exhibited G0/G1 phase arrest after beuing treated with different concentrations of NaBu (0, 0.125, 0.25, 0.5, 1, and 2 mmol/L) for 3 days. Cells were stained with PI and analyzed by FCM. **b** The adapted cells adherent cultured in 0.5% serum also exhibited G0/G1 phase arrest after treated as same as **a** (*p < 0.05 and **p < 0.01)

To further unravel mechanisms responsible for proliferation slow-down post-NaBu treatment, cell cycle analysis was performed using FCM. 1 × 10^5^ FCHO/IL-24 cells were seeded in 6-well plates and treated with different concentrations of NaBu (0, 0.125, 0.25, 0.5, 1, and 2 mmol/L) for 3 days in media containing 10% and 0.5% serum. As shown in Fig. 3, percentage of cells in G0/G1 phase increased significantly with increasing NaBu concentration. Under 10% serum culture, the percentage of G0/G1 phase cells in 2 mmol/L NaBu treatment group increased from 37.60 ± 3.75% (without NaBu) to 55.02 ± 5.46% and the highest increase rate was 46 ± 8% (**p < 0.01) (Fig. 3a), compared to those of untreated control group. In 0.5% serum culture, the percentage of G0/G1 phase cells increased from 46.06 ± 1.37% (untreated) to 65.46 ± 1.97% (2 mmol/L NaBu) with an increase rate of 42 ± 8% (**p < 0.01) (Fig. 3b). However, under both 10% serum and 0.5% serum culture, the percentage of S phase decreased significantly (**p < 0.01) and no significant change was observed in G2/M phase (*p* > 0.05).

### NaBu had no obvious effect on inducing apoptosis of FCHO/IL-24 cells

**Fig. 4:**
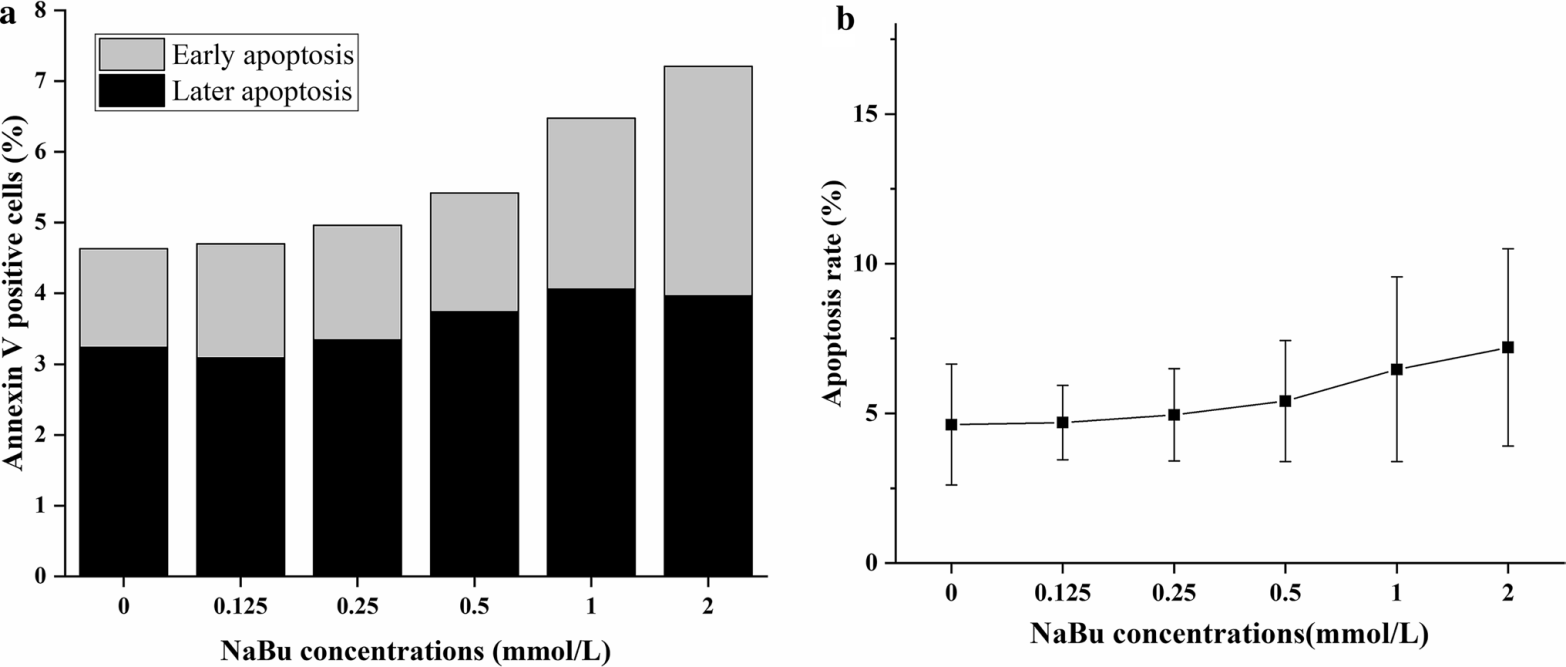
NaBu had no significant effect on inducing apoptosis of FCHO/IL-24 cells. **a** The effect of NaBu on FCHO/IL-24 cell apoptosis (1 × 10^5^ cells/well in 6-well plate) across various treatment dose (0, 0.125, 0.25, 0.5, 1, and 2 mmol/L) for 3 days, stained by Annexin V-FITC, and checked by FCM. Annexin V staining helped differentiate early apoptosis cells (Annexin V^+^ and PI^−^) and late apoptosis cells (Annexin V^+^ and PI^+^). **b** With increasing NaBu concentrations, apoptotic cell percentage slightly increased, but the change is not statistically significant (*p* > 0.05)

To investigate the effects of NaBu treatment on apoptosis, 1 × 10^5^ adapted FCHO/IL-24 cells were plated in each well of 6-well plates, treated by different concentrations of NaBu as mentioned above for 3 days in DMEM/F12 with 10% serum, stained by Annexin-V and analyzed by flow cytometry. Figure 4 showed that high NaBu concentrations seemed to promote apoptosis modestly in engineered cells. As NaBu concentration increased from 0 to 2 mmol/L, apoptotic cells percentages were 4.63 ± 2.02%, 4.70 ± 1.24%, 4.96 ± 1.54, 5.41 ± 2.01%, 6.47 ± 3.08%, and 7.21 ± 3.29%, respectively. However, the increase was not significant (*p* > 0.05), indicating that NaBu that does not significantly promote cell apoptosis in addition to the benefits of enhancing IL-24 production.

### NaBu affected glucose, lactate and ammonia concentrations in the cell culture supernatant

**Fig. 5:**
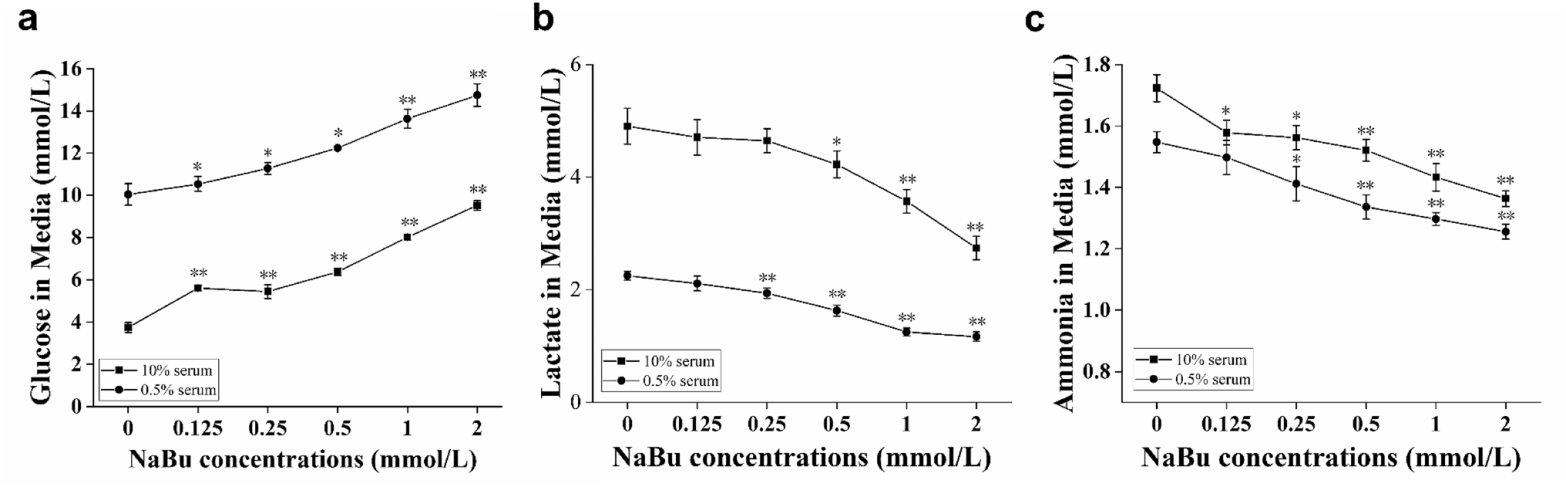
The concentrations of glucose, lactate and ammonia in the culture supernatant of FCHO/IL-24 cells were affected by NaBu treatment. **a–c** With increased NaBu dose, the concentrations of glucose increased and those of lactate and ammonia decreased under both 10% serum and 0.5% serum adherent culture conditions. 650 and 2000 adapted cells were seeded in each well of 96-well plate in 200 μL DMEM/F12 with 10% and 0.5% serum, respectively

NaBu treatment can not only induce G0/G1 phase arrest in FCHO/IL-24 but also inhibit cell proliferation and up-regulate rhIL-24 expression level. It is likely that NaBu treatment triggered alterations in metabolic pathways in favor of enhanced protein production. Glucose, lactate and ammonia concentrations are closely related to cell proliferation and protein expression thus could play important roles in altered cell signaling following NaBu treatment. To validate our hypothesis, we treated adapted FCHO/IL-24 cells with varying NaBu concentrations (0, 0.125, 0.25, 0.5, 1, and 2 mmol/L) for 3 days in either 10% or 0.5% serum medium and tested concentrations of glucose, lactate and ammonia in the culture supernatant by Spectrophotometry. With a higher dose of NaBu treatment, glucose concentration increased significantly (**p < 0.01) while concentrations of lactate and ammonia reduced evidently (**p < 0.01), and the trends were similar between 10% and 0.5% serum groups (Fig. 5a–c). As glucose was the primary carbon source in cell culture, the higher the cell proliferation rate, the faster the glucose consumption rate, and the less glucose remained in the supernatant. As cell proliferation was negatively affected by NaBu in a dose-dependent manner, glucose concentration increased with the NaBu dose. In contrast, lactate (by-products of carbohydrate metabolism) and ammonia (product of glutamine degradation) evidently decreased with increasing NaBu dose. At the same NaBu treatment concentration, glucose concentration in the supernatant was higher while the lactate and ammonia concentrations were lower in 0.5% serum group than in those of 10% serum group, which indicated that lower serum and NaBu treatment would synergistically reduce aerobic respiration metabolism in culture.

### NaBu treatment altered tumor and immune-related signaling pathways

**Fig. 6:**
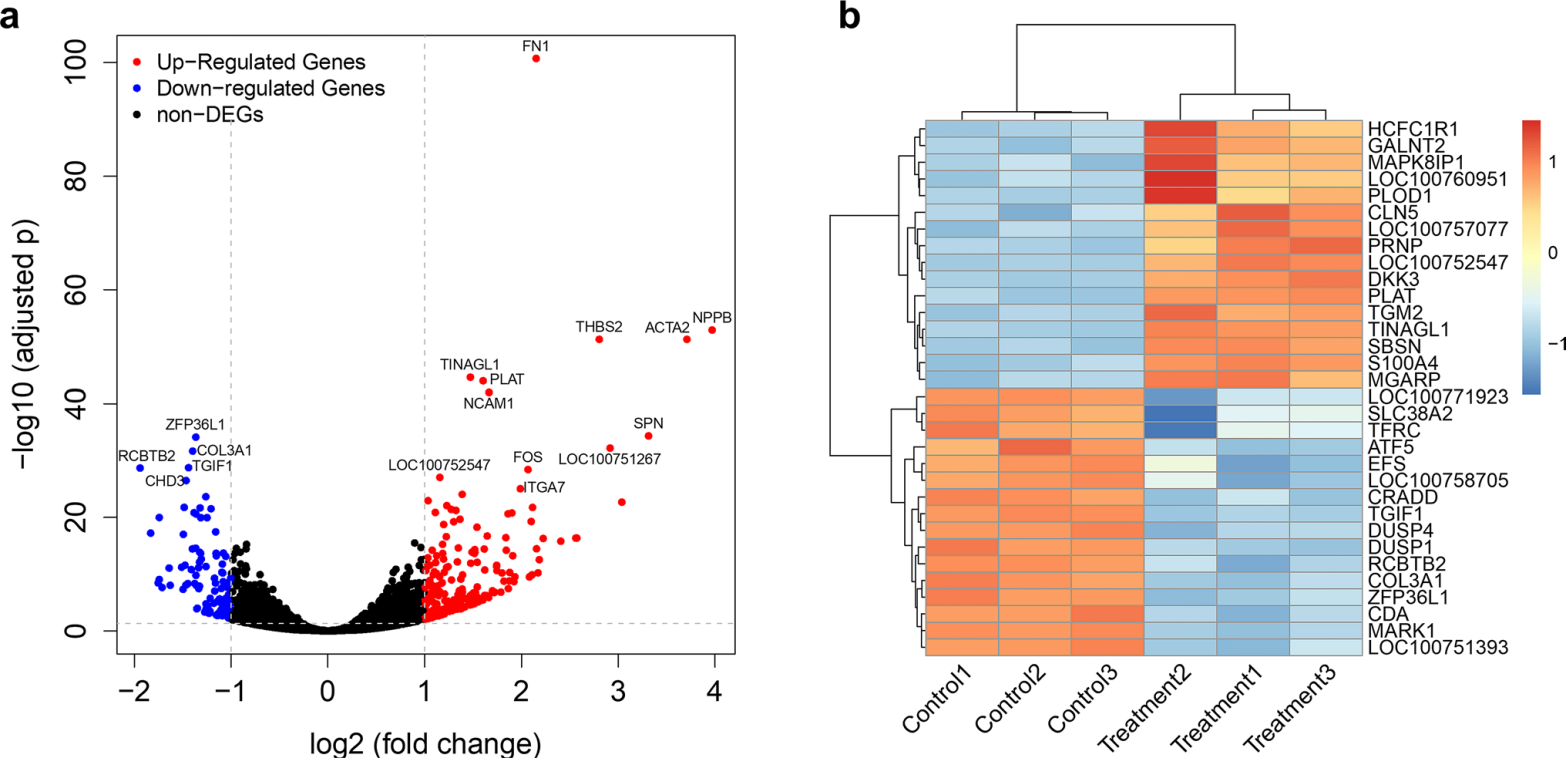
Differential gene expression analysis. **a** Volcano plot for DEGs between control and treatment groups. Red dots stand for significantly up-regulated genes in the treatment group, blue dots stand for significantly down-regulated genes, and black dots stand for genes not significantly different between the control and treatment group. **b** Each row represents a gene, and each column represents a sample. The X-axis shows sample names and the clustering result of six samples, and the Y-axis shows DEGs and the clustering result of these genes. The color in the heatmap corresponds to Z scores of gene expression level Log2 (FPKM + 1)

To further investigate the downstream signaling of NaBu treatment, we utilized transcriptomics and conducted differential expression analysis. 226 significantly up-regulated genes and 93 significantly down-regulated differential genes were identified between control and treatment groups, as shown in Fig. 6a. Top 20 significant differentially expressed genes (DEG) were listed in Tables 1 and 2, respectively. These changes in gene expression at the transcriptional level may be associated with observed phenotypic changes in FCHO/IL-24 cells after NaBu treatment. Although the IL-24 gene was not significantly differentially expressed between the two groups (Log2FC = 0.05, adjusted *p* = 0.93), many DEGs are closely related to IL-24. It was reported that IL-24 expression during terminal differentiation in human melanoma cells is regulated predominantly at a posttranscriptional level [25].

**Table 1:** Top 20 significantly up-regulated genes of FCHO/IL-24 cells treated by NaBu

Gene ID	P_adj	Log2(FC)	Symbol	Description
100,751,189	1.83E-101	2.152	FN1	Fibronectin 1
100,773,766	1.22E-53	3.970	NPPB	Natriuretic peptide B
100,752,022	5.24E-52	2.804	THBS2	Thrombospondin 2
100,758,575	5.24E-52	3.709	ACTA2	Actin, alpha 2, smooth muscle, aorta
100,760,266	2.34E-45	1.472	TINAGL1	Tubulointerstitial nephritis antigen like 1
100,750,775	1.04E-44	1.604	PLAT	Plasminogen activator, tissue type
100,770,418	1.08E-42	1.665	NCAM1	Neural cell adhesion molecule 1
100,752,546	4.58E-35	3.313	SPN	Sialophorin
100,751,267	6.38E-33	2.916	LOC100751267	Intercellular adhesion molecule 2
100,689,418	3.82E-29	2.068	FOS	Fos proto-oncogene, AP-1 transcription factor subunit
100,752,547	9.25E-28	1.157	LOC100752547	Histone H1.0
100,773,038	9.36E-26	1.989	ITGA7	Integrin subunit alpha 7
100,759,289	8.85E-25	1.388	TGM2	Transglutaminase 2
100,767,109	1.14E-23	1.035	DKK3	Dickkopf WNT signaling pathway inhibitor 3
103,160,317	2.17E-23	3.037	LOC103160317	ankyrin repeat Domain-containing protein 26-like
100,768,152	8.51E-23	1.229	SBSN	Suprabasin
100,769,003	1.74E-22	2.114	WNT4	Wnt family member 4
100,752,370	4.00E-22	1.274	P3H2	Prolyl 3-hydroxylase 2
100,770,532	5.74E-22	1.323	S100A4	S100 calcium binding protein A4
100,762,499	1.39E-21	1.110	MTSS1	MTSS1, I-BAR domain containing

**Table 2:** Top 20 significantly down-regulated genes of FCHO/IL-24 cells treated by NaBu

Gene ID	P value	Log2(FC)	Symbol	Description
100,759,015	7.37E-35	− 1.364	ZFP36L1	ZFP36 ring finger protein like 1
100,753,589	2.16E-32	− 1.396	COL3A1	Collagen type III alpha 1 chain
100,774,130	1.75E-29	− 1.439	TGIF1	TGFB-induced factor homeobox 1
100,750,578	2.04E-29	− 1.94	RCBTB2	RCC1 and BTB domain-containing protein 2
100,755,630	3.23E-27	− 1.464	CHD3	Chromodomain helicase DNA binding protein 3
100,756,233	2.31E-24	− 1.261	DUSP4	Dual specificity phosphatase 4
100,773,206	1.78E-22	− 1.484	NSD1	Nuclear receptor binding SET domain protein 1
100,762,795	2.19E-22	− 1.32	ARHGEF6	Rac/Cdc42 guanine nucleotide exchange factor 6
100,757,648	3.11E-22	− 1.207	MARK1	microtubule affinity Regulating kinase 1
100,771,923	1.52E-21	− 1.383	LOC100771923	Laminin subunit alpha-2
100,751,393	2.51E-21	− 1.363	LOC100751393	Schlafen family member 12-like
100,752,521	1.05E-20	− 1.742	TSPOAP1	TSPO-associated protein 1
100,769,474	1.09E-20	− 1.248	DUSP1	dual specificity phosphatase 1
100,754,972	1.10E-20	− 1.313	CDA	Cytidine deaminase
100,769,320	3.51E-18	− 1.16	ATF5	activating transcription factor 5
100,771,140	5.87E-18	− 1.831	C3AR1	Complement C3a receptor 1
100,755,135	9.87E-18	− 1.493	ZNF469	Zinc finger protein 469
100,753,942	2.62E-15	− 1.365	PRRX1	Paired related homeobox 1
100,769,451	3.46E-15	− 1.400	DNAJC22	DnaJ heat shock protein family (Hsp40) member C22
100,756,504	1.39E-14	− 1.324	PHF19	PHD finger protein 19

A couple DEGs are of particular interest. DKK3 is an inflammatory suppressor and its mRNA level decreased after dextran sodium sulfate (DSS) induction in the free fatty acid receptor 2 (FFAR2)-deficient colon tumor mice. FFAR2 is an epigenetic tumor suppressor that could be activated by butyrate and is required for butyrate to suppress HDAC expression and hypermethylation of inflammation suppressors [26]. Thus, we speculate that NaBu-FFAR2-DKK3 may be activated sequentially, which is consistent with the upregulation of DKK3 (Log2FC = 1.035, adjusted *p* = 1.14E-23) in Table 1. Both DKK3 and IL-24 are differentially expressed in pig ovarian follicular atresia as regulatory candidates of pig granulosa cell atresia [27]. It is well-known that both IL-24 and COL3A1 involved in migration and invasion. For example, methylmercury could reduce the proportion of cells in G1 phase and cell migration and invasion capacities, and IL-24 is significantly upregulated while COL3A1 is significantly downregulated in methylmercury-treated group [28]. Another study found that microneedling therapy in a human three-dimensional skin model composed of epidermal keratinocytes, dermal fibroblasts and collagen–elastin matrix could induce downregulation of IL-24 and upregulation of COL3A1 [29]. Therefore, after NaBu treatment, when COL3A1 expression was significantly downregulated (Log2FC = -1.396, adjusted p = 2.16E-32) in Table 2, the enhancement of IL-24 production was expected.

### Functional annotation and pathway enrichment

**Fig. 7:**
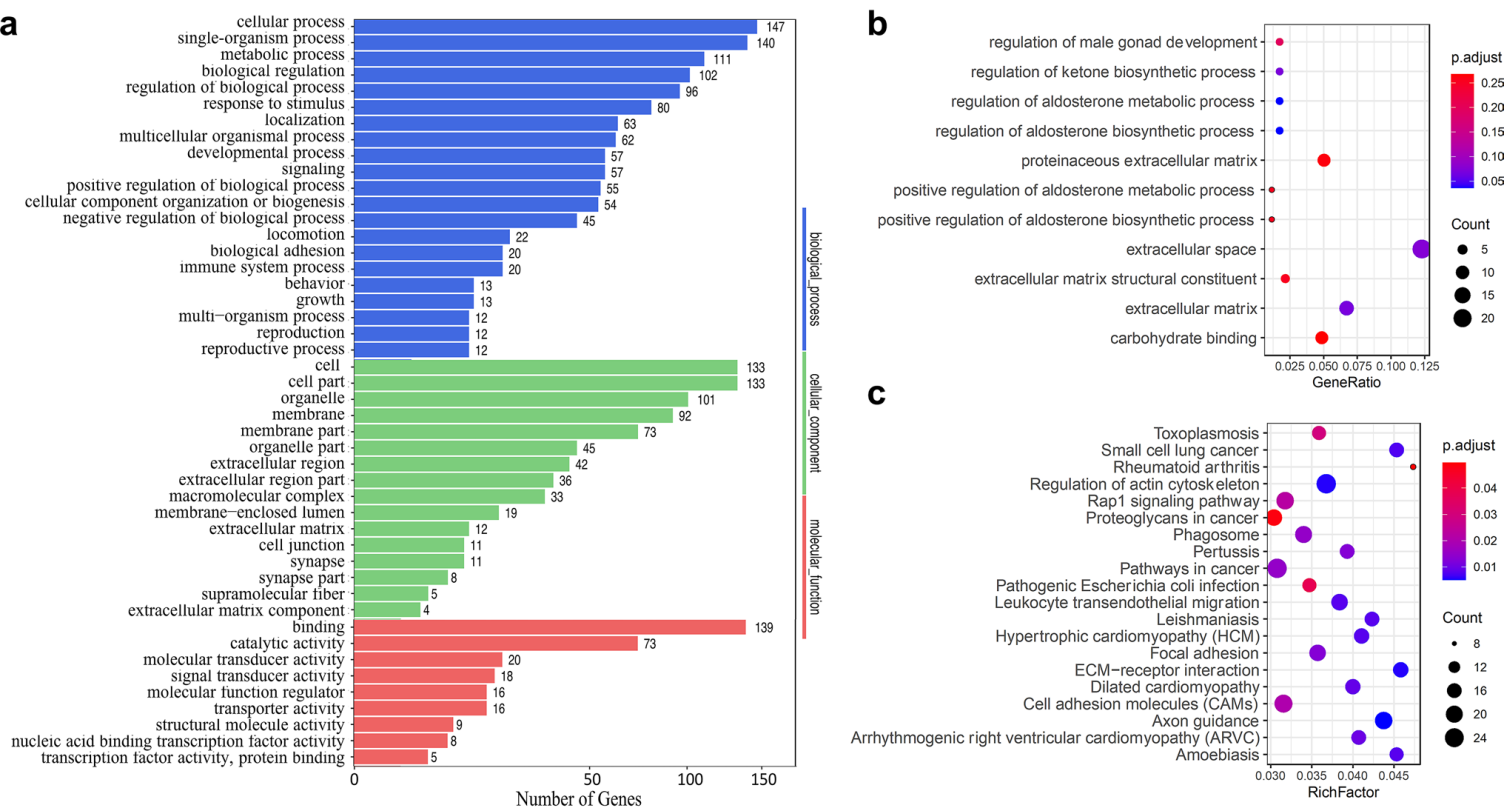
GO functional annotation and enrichment. **a** DEGs in each level-2 GO term with GO functional annotations. **b** GO terms with adjusted *p* < 0.3 were demonstrated. Only “regulation of aldosterone metabolic process” (adjusted *p* = 0.036) and “regulation of aldosterone biosynthetic process” (adjusted *p* = 0.036) are considered as significantly enriched. **c** KEGG pathways with adjusted *p* < 0.05 were demonstrated

The number of DEGs in each level-2 GO term was demonstrated in Fig. 7a, with brief functional annotations of these genes. GO enrichment analysis of DEGs was also performed, only 2 GO terms are significantly enriched (adjusted *p* ≤ 0.05), namely “regulation of aldosterone metabolic process” and “regulation of aldosterone biosynthetic process”. Since the number of significantly enriched GO terms are too small, all Go terms with adjusted *p* ≤ 0.3 were illustrated in Fig. 7b. Many studies have consistently shown that aldosterone activates cells in the innate and adaptive immune systems [30], for example, aldosterone may stimulate IL‐6 production [31]. Only significantly enriched KEGG pathways with adjusted *p* < 0.05 were demonstrated in Fig. 7c. Several pathways are related to cancer, including “Small cell lung cancer”, “Proteoglycans in cancer”, and “Pathways in cancer”. And several pathways are related to immune, such as autoimmune disease pathways “Leukocyte transendothelial migration” and “Rheumatoid arthritis”.

## Discussion

To realize full potential of IL-24 as an anticancer therapeutic target, it is important to improve rhIL-24 production in mammalian cell systems. In this study, the protein expression level of rhIL-24 in the culture supernatant of the site-specified engineered cell line FCHO/IL-24 was significantly improved by NaBu treatment under different culture conditions (10% and 0.5% serum adherent culture, 0.5% serum suspension culture). NaBu treatment inhibited cell proliferation, induced G0/G1 cell arrest and influenced metabolite concentrations without triggering higher levels of apoptosis. RNA-seq analysis reveals that NaBu treatment has almost no effect on the mRNA expression level of rhIL-24. But NaBu treatment can affect several tumor and immune-related pathways in FCHO/IL-24 cells, which may indirectly stimulate the secretion of rhIL-24 protein.

In previous studies, 1 mmol/L was commonly used as the final concentration of NaBu to treat engineered CHO cell lines, such as thyrotropin [13], prolactin [12], and t-PA [9], and the expression levels were increased by 330%, 200%, and 212%, respectively. There were also reports using much higher concentrations of NaBu to treat recombinant CHO cell line, such as 5 mmol/L for MGpUK-5 cells (producing single-chain urokinase-type PA, scu-PA) in the serum-free perfusion culture [32], and 8 mmol/L for CHO cells producing cB72.3 IgG4 monoclonal antibody [33]. Our results showed that different concentrations of NaBu could increase the expression of rhIL-24 in FCHO/IL-24 cells independent of culture conditions such as adherent or suspension culture, or high (10%) vs low (0.5%) serum. After 3 days of 1 mmol/L NaBu treatment, rhIL-24 expression levels were increased by 119.94 ± 1.5% (**p < 0.01), 57.49 ± 2.4% (**p < 0.01), and 20.17 ± 3.03% (**p < 0.01) under 10% serum adherent culture, 0.5% serum adherent culture, and 0.5% serum suspension culture, respectively. The beneficial effect of using high concentrations of NaBu on rhIL-24 expression was compromised by its detrimental impact on cell growth and proliferation. For example, in 10% serum adherent culture, when treated with 1 and 2 mmol/L NaBu for 3 days, FCHO/IL-24 proliferation was inhibited (9 ± 3% and 18 ± 4%, respectively), contributing to an overall modest increase of rhIL-24 in the culture supernatant (3.56 ± 0.09 and 4.21 ± 0.65 ng/mL). This trend is also consistent if the adherent culture serum was decreased to 0.5%. Under 0.5% suspension culture, the concentrations of rhIL-24 were 4.39 ± 0.15 and 4.80 ± 0.30 ng/mL, respectively, but the inhibition rate on cell proliferation was both 12 ± 1%. Thus, 1 mmol/L and 2 mmol/L were selected as the optimum concentrations of NaBu to treat FCHO/IL-24 cells under adherent culture and suspension culture, respectively.

It has been reported that NaBu can promote G0/G1 phase arrest, and affect energy metabolism and protein synthesis in engineered CHO cells [9, 13]. Our flow cytometry results showed that at the same treatment time point, the higher the NaBu concentration, the higher the percentage of cells in G0/G1 phase, while the percentage of S phase cells decreased significantly. We also checked if apoptosis was induced as reported previously [34], but percentages of apoptotic cells did not exceed 8% when the NaBu concentration was 1 mmol/L, and only increased by 1.84 ± 1.06%. All these results suggested that contrary to other studies, NaBu treatment did not cause severe apoptosis, but contributed to the decrease of relative cell viability via G0/G1 phase arrest.

So far, the cellular and molecular mechanisms responsible for phenotypic changes in engineered CHO cells following NaBu treatment were not well understood. For the CHO DP-12 cells producing anti-IL-8 antibody, NaBu treatment exerted a strong and rapid impact on DNA-methylation by CpG-microarray, DNA modification by bisulfite sequencing, and significantly altered the abundance of 118 proteins or subunits by 2DE-gels and fast high-resolution ESI–MS [35]. When EPO-producing CHO cells were treated with NaBu in batch culture, autophagy was induced, which may act as a positive survival mechanism in response to NaBu treatment. At the same time, apoptosis was also induced and the relationship between autophagy and apoptosis remain an interesting question [36]. As IL-24 is a cytokine, it is plausible that the synthesis and secretion of IL-24 are deeply involved in immune regulation. In addition to tumor and I mmune-related pathways mentioned above, the DEGs were also involved in pathways of infectious diseases, such as “Amoebiasis”, “Leishmaniasis”, “Pertussis”, “Toxoplasmosis” and “Pathogenic Escherichia coli infection”, suggesting that signaling post-NaBu treatment is closely immune-related.

In short, under different serum concentrations and culture methods, NaBu could inhibit FCHO/IL-24 proliferation in a dose-dependent manner, but simultaneously increase rhIL-24 concentration and yield per cell. Our results suggest that this was achieved by inducing G0/G1 arrest, keeping cells at high productivity while illiciting limited induction on apoptosis. These results implied that NaBu can be used as an additive to serum-free medium for suspension culture to further facilitate the production of rhIL-24 on an industrial scale as a promising anti-cancer therapeutic.

## Materials and methods

### Reagents

NaBu (B5887-250MG) were purchased from Sigma-Aldrich Chemical Corporation (St. Louis, MO, USA). Cell culture media, Dulbecco’s modified Eagle's medium/Ham’s F12 (DMEM/F12, 12,400–024) was purchased from Life Technologies Corporation (NY 14,072, USA). Human interleukin-24 ELISA kit (F01531) was purchased from Xitang Biotechnology (Shanghai, China). Annexin V-FITC Apoptosis Detection Kit (KGA107) was purchased from Kaiji Biotechnology (Nanjing, China). Glucose detection kit (E1010) was purchased from Applygen Technologies (Beijing, China). Lactic acid detection kit (A019-2) and urea nitrogen kit (C013-2) were purchased from Jiancheng Biological Company (Nanjing, China).

### Cell culture

The site-specific-integrated cell line, Flp-In-CHO/IL-24 (FCHO/IL-24), was prepared by our research group and maintained in Ham’s F12 medium supplemented with 10% FBS, 100 μg/ml penicillin/streptomycin, 2 mmol/L L-glutamine, HEPES buffer and hygromycin (400 μg/ml) [4]. After adaptation to serum-free suspension culture, the FCHO/IL-24 cells could grow well in DMEM/F12 under different culture conditions used in this study, such as 10% serum adherent culture, 0.5% serum adherent culture and 0.5% serum suspension culture [23]. In suspension culture, the cells were cultured in a 125 mL shake flask containing 20 mL culture medium of 0.5% serum DMEM/F12 at 119 rpm, cell seeding density of 5 × 10^5^ cells/mL, and 37 °C, in a 5% CO_2_ and 95% humidity atmosphere.

### Sodium butyrate treatment

In adherent culture, under 10% and 0.5% serum conditions, the numbers of cells seeded in 96-well plate were 650 and 1500 per well, respectively, and 1 × 10^5^ per well in 6-well plate. After 24 h, the medium was changed to the medium containing different concentrations of sodium butyrate (0, 0.125, 0.25, 0.5, 1, and 2 mmol/L), and the serum concentration remained unchanged. If the experiment exceeds 3 days, the culture medium was changed every 3 days to maintain the sodium butyrate concentration. In suspension culture, the cell seeding density was 5 × 10^5^ cells/mL, and different final concentrations of sodium butyrate as mentioned above was added at the same time.

### Cell viability assay

Cell viability was assessed by MTT assay. Cells were seeded in 96-well plates for 24 h at 37 °C. On the day after treatment, the medium was removed and 5 mg/ml MTT was added to each well. The cells were maintained at 37 °C for 4 h, then, 150 µl of dimethyl sulfoxide (DMSO) was added to each well and mixed thoroughly. The absorbance was read on a Bio-Rad microplate reader Model 550 (Hercules, CA, USA) at 490 nm. The MTT absorbance of the untreated control cells was set to 1 to calculate the relative number of viable cells. The experiments were repeated at least 3 times to ensure reproducibility and statistical significance.

### ELISA

The ELISA reaction to detect concentrations of rhIL-24 was conducted in 96-well plates according to the manufacturer’s instructions. Briefly, samples (100 µl) were diluted, added to 96-well plates and incubated at 37 °C for 2 h. The plate was incubated with a biotinylated antibody against IL-24 for 1 h at 37 °C and then with HRP-streptavidin for 30 min at room temperature. The reaction was developed with the addition of TMB peroxidase substrate and stopped with 1 N H_2_SO_4_. The optical density (OD) values were read on a Bio-Rad microplate reader Model 550 (Hercules, CA, USA) at 450 nm. The concentrations of IL-24 were determined via comparison with the standard curve.

### Cell cycle

FCHO/IL-24 cells were seeded at 1 × 10^5^ cells/well in 6-well plates and treated with NaBu at different concentrations (0, 0.125, 0.25, 0.5, 1 and 2 mmol/L) the following day. On day 3 after treatment, the cells were harvested following trypsinization without ethylenediaminetetraacetic acid (EDTA), washed with cold PBS and fixed by suspending the cells in 1 ml stationary liquid (30% PBS and 70% ethanol) at − 20 °C. After 3 days, the fixed cells were washed and resuspended in 300 µl PI (50 µg/ml) and 10 µl RNase A (10 mg/ml). Then, the samples were incubated at 37 °C for 30 min in the dark and DNA content was analyzed by flow cytometry (FCM).

### Apoptosis assay

Cell apoptosis was detected via the Annexin V binding assay and FCM. FCHO/IL-24 cells were seeded and treated with NaBu in the same way as the cell cycle distribution assay. On day 3 after treatment, the cells were stained with FITC-labeled Annexin V and propidium iodide (PI) according to the manufacturer's instructions (KGA105-50, Annexin V-FITC apoptosis detection kit; Nanjing KeyGen Biotech, Nanjing, China) and FCM was performed immediately after staining.

### Detection of glucose, lactate and ammonia concentrations in cell culture supernatant

The concentrations of glucose, lactic acid and ammonia in the cell culture supernatant were determined according to kit instructions. Glucose concentration in the culture medium was quantified by an assay kit (E1010) using the gluocose oxidase (GOD) method. 5 μL standard sample or test sample were added to 96-well plates, reacted with 195 μL working solution mixed by reagent R1 and R2 at 37 °C for 20 min, and then OD550 values were read. The concentration of ammonia was checked by a urea nitrogen kit (C013-2) and the 5 mmol/L ammonium chloride was as the standard conrol. After 20 μL standard or sample in a centrifuge tube was mixed with 1 mL phenolic chromogenic solution and 1 mL alkaline sodium hypochlorite at 37 °C for 10 min, 300 μL reaction solution were added into 96-well plates to read OD640 values. In the lactate concentration detection kit (A019-2), 20 μL standard or sample were reacted with 1 mL enzyme working solution and 1 mL chromogen solution at 37 °C for 10 min, then stopped by stop solution and the OD530 values were read. All the test samples were collected in three independent experiments. The concentrations of glucose, lactic acid and ammonia in the cell culture supernatant were all determined via comparison with the standard curves.

### RNA-seq analysis

After 1.5 × 10^6^ FCHO/IL-24 cells were seeded in 10 cm dishes with 10 mL 10% serum DMEM/F12 for 24 h, the cells were equally divided into two groups, namely the treatment group, treated by 1 mmol/L NaBu for 3 days, and the control group with PBS. Total RNA was extracted from each group using TRIzol Reagent (Invitrogen, Carlsbad, CA) by routine protocols. RNA quality and purity were assessed using Agilent 2100 Bioanalyzer (Agilent Technologies, Santa Clara, CA) and NanoDrop 2000 Spectrophotometer (Thermo Fisher, Waltham, MA), to make sure only high-quality RNA samples were used for library preparation. The transcriptome libraries were made using the Illumina TruSeq RNA sample preparation Kit (Illumina, San Diego, CA) according to the manufacturer’s instructions. The libraries were sequenced on Illumina Hiseq X10 platform (Illumina, San Diego, CA), and 150-bp paired-end reads were generated.

Quality control of raw reads was performed using fastp [37] and Trimmomatic [38]. Clean reads were obtained after trimming reads with adapters and removing low-quality reads. The clean reads were aligned to the reference genome using Hisat [39], and the reference-based assembly of transcripts was performed using StringTie [40] to detect novel transcripts. Then Bowtie2 [41] was applied for aligning clean reads to sequences of all transcripts, and RSEM [42] was applied for the quantification of transcripts and genes by analyzing alignment output of bowtie2. The statistical analyses for differentially expressed genes (DEG) were performed using the R package DEseq2 [43]. The fold change (FC) of the gene between the two groups was calculated, and the false discovery rate (FDR) was used for p-value adjustments. Genes with adjusted *p* ≤ 0.001 and |Log2FC|≥ 1 were considered as significant DEGs. Hierarchical clustering of top DEGs in six samples was performed using the R package pheatmap.

For functional annotation and classification, the enrichment analysis of the significant DEGs in gene ontology (GO) and Kyoto Encyclopedia of Genes and Genomes (KEGG) database was performed using the R package cluster Profiler [44]. Those GO terms and KEGG Pathways with FDR adjusted *p* ≤ 0.05 were considered as significantly enriched.

### Statistical analysis

For MTT, cell count, ELISA, FCM and RNA-seq, the experiments were performed at least three times. Statistical comparisons between groups were performed with an unpaired Student’s *t* test using SPSS software version 19. Standard curve fitting and sample concentration calculation were performed on the ELISA data and the supernatant metabolite detection data using Origin 2018 software. *p < 0.05 or **p < 0.01 was considered to indicate a statistically significant result.

## Data Availability

The datasets generated and analyzed in this study are available from the corresponding author upon reasonable request.
